# Comparative effectiveness of a mindfulness-based intervention (M-Body) on depressive symptoms: study protocol of a randomized controlled trial in a Federally Qualified Health Center (FQHC)

**DOI:** 10.1186/s13063-022-07012-2

**Published:** 2023-02-17

**Authors:** Inger Burnett-Zeigler, Elayne Zhou, Jennifer H. Martinez, Katelyn Zumpf, Lynette Lartey, Judith T. Moskowitz, Katherine L. Wisner, Thomas McDade, C. Hendricks Brown, Jacqueline Gollan, Jody D. Ciolino, Jacob M. Schauer, Lucia C. Petito

**Affiliations:** 1 Asher Center, Department of Psychiatry and Behavioral Sciences, Feinberg School of Medicine, Northwestern University, 676 N St. Clair St, Chicago, IL 60611 USA; 2 Division of Biostatistics, Department of Preventive Medicine, Feinberg School of Medicine, Northwestern University, Chicago, IL USA; 3 Department of Medical Social Sciences, Feinberg School of Medicine, Northwestern University, Chicago, IL USA; 4 Department of Anthropology, Weinberg College of Arts and Sciences, Northwestern University, Chicago, IL USA

**Keywords:** Mindfulness, Depression treatment, Disparities, Health equity, Community health

## Abstract

**Background:**

Mindfulness-based interventions have been shown to improve psychological outcomes including stress, anxiety, and depression in general population studies. However, effectiveness has not been sufficiently examined in racially and ethnically diverse community-based settings. We will evaluate the effectiveness and implementation of a mindfulness-based intervention on depressive symptoms among predominantly Black women at a Federally Qualified Health Center in a metropolitan city.

**Methods:**

In this 2-armed, stratified, individually randomized group-treated controlled trial, 274 English-speaking participants with depressive symptoms ages 18–65 years old will be randomly assigned to (1) eight weekly, 90-min group sessions of a mindfulness-based intervention (M-Body), or (2) enhanced usual care. Exclusion criteria include suicidal ideation in 30 days prior to enrollment and regular (>4x/week) meditation practice. Study metrics will be assessed at baseline and 2, 4, and 6 months after baseline, through clinical interviews, self-report surveys, and stress biomarker data including blood pressure, heart rate, and stress related biomarkers. The primary study outcome is depressive symptom score after 6 months.

**Discussion:**

If M-Body is found to be an effective intervention for adults with depressive symptoms, this accessible, scalable treatment will widely increase access to mental health treatment in underserved, racial/ethnic minority communities.

**Trial registration:**

ClinicalTrials.gov NCT03620721. Registered on 8 August 2018.

## Introduction

### Background

Socio-economically disadvantaged adults, including racial/ethnic minorities, are at greater risk for a depressive episode in the last year [1–5]. Stress is an important factor in the development and chronicity of depressive symptoms [6, 7]. Psychosocial stress is associated with increased concentrations of C-reactive protein (CRP), IL-6, and IL-1β [8–10], inflammatory cytokines that have been consistently linked to depression [11, 12]. Black adults are disproportionally exposed to psychosocial stressors such as economic deprivation, unemployment, racism, and exposure to violence [6, 7, 13–15]. The disproportionate burden of stress experienced by Black adults places them at greater risk for depression.

Depression among socio-economically disadvantaged, Black adults is not adequately treated. Black adults are 40–60% less likely than those who are White to receive mental health treatment [16–18] and less likely to receive adequate treatment [19, 20]. Negative attitudes toward depression treatment [21], stigmatizing beliefs about mental illness and treatment [22–24], shame and self-blame associated with treatment-seeking [25], and higher levels of medical mistrust [26] are barriers to receiving treatment among Black adults.

Those who do receive depression treatment are more likely to find it in primary care versus specialty mental health clinics [27–29]. Federally Qualified Health Centers (FQHC) are safety net providers of primary, preventive, and specialty outpatient healthcare, including mental health/substance abuse, to individuals in underserved communities. Integration of evidence-based depression treatments in FQHCs is economically justified, likely to reduce systemic and psychological barriers, and may facilitate access to and engagement in mental health care [30–32].

The US Department of Health and Human Services Agency for Healthcare and Research Quality recommends mindfulness-based interventions in their guidelines for non-pharmaceutical management of depression in adults [33]. Mindfulness-based Stress Reduction (MBSR), an 8-week group intervention that teaches mindfulness skills through a range of formal and informal practices including mindfulness of breath, bodily sensations, sounds, thoughts, and everyday activities, is one of the most widely used mindfulness interventions [34]. Mindfulness-based interventions have collectively been shown to improve physical and mental health [35–38] and reduce depression symptom severity (Cohen’s *d* ~.59–.69) [37, 39]. MBSR specifically has been associated with improved stress biomarkers including reduced blood pressure [40–45], reduced inflammatory response [46, 47], and increased immune response [48].

There is a paucity of research examining the effectiveness of mindfulness-based interventions among socio-economically disadvantaged, racial/ethnic minority adults. A recent systematic review examining the socio-economic and demographic characteristics of participants in mindfulness-based randomized controlled trials found an over-representation of participants who were White, held a college degree, were employed, and had an annual income of over $40,000 [49]. Only one randomized controlled trial has been conducted in the USA to test the effectiveness of MBSR or Mindfulness-Based Cognitive Therapy (MBCT) in improving psychological outcomes among Black adults in a community-based setting [50]; however, effectiveness data from this study have not been published [50, 51].

In a meta-analysis of acceptance and mindfulness-based treatments with underserved populations (*N*= 35) [52], only two studies evaluated MBSR or MBCT with majority racial/ethnic minority adults [53, 54]. One of the two studies found that participation in MBSR among socio-economically disadvantaged, racial/ethnic minority adults was associated with significant changes in reported psychological outcomes compared to a no-contact control (Hedge’s g = .67) [53]. More recently, another study evaluated the effectiveness of MBCT in improving psychological outcomes of racial/ethnic and sexual minority women and demonstrated improvement in depressive and stress symptoms in addition to satisfactory feasibility and acceptability of the intervention [55].

The minority stress theory [56], based on stress and coping theory, frames our proposed research. Stress and coping theory states that stress occurs when the demands of a situation are perceived as exceeding the resources available and coping is the cognitive and behavioral efforts put forth to deal with the stressful situation [57]. The minority stress theory distinguishes the excess stress that individuals belonging to stigmatized social categories, including categories related to socioeconomic status, race/ethnicity and gender, experience that likely have physical and mental effects. This theory accounts for the stress associated with personal events (i.e., trauma) and environmental and social conditions.

Appropriately tailored interventions and service delivery systems are urgently needed to increase access to and engagement in evidence-based mental health treatments among individuals who are at greater risk for depression but have less access to treatment. In this paper, we will describe the rationale behind, design, and implementation of a randomized controlled trial to evaluate the comparative effectiveness of a mindfulness-based intervention on depressive symptoms among predominantly Black women in a Federally Qualified Health Center network.

### Prior work

The PI led a series of pilot studies that demonstrated the acceptability, feasibility, and impact on primary outcomes of a mindfulness-based intervention (M-Body) among Black women with depressive symptoms in an FQHC. M-Body is a version of the evidence-based Mindfulness-based Stress Reduction (MBSR) program that has been tailored for the FQHC patient population and healthcare service delivery setting [58].

Women ages 18–65 with a positive depression screen were recruited from a FQHC on the South Side of Chicago. Interested, eligible participants were invited to participate in the 8-week, 90-min-per-session, M-Body group intervention. Depression (Inventory of Depressive Symptomatology – Clinician Rated and Self-Report and Mini International Neuropsychiatric Interview 6.0, Quick Inventory of Depressive Symptomatology – Clinician Rated), well-being (Ryff Psychological Well-Being Scale – 42 item), mindfulness (Five Facet Mindfulness Questionnaire), stigma (Depression Self Stigma Scale), functioning (World Health Organization Disability Adjustment Scale 2.0 – 12 item), and stress (Perceived Stress Scale) were assessed as primary outcomes at baseline and 8 and 16 weeks.

In total, 161 women were referred, 114 screened, 102 eligible, and 74 enrolled in the single-arm M-Body intervention. All participants except one were of Black race. The mean age was 49 years, the majority were single (58%), 61% had a personal income of $19,999 or less, 28% were unemployed, 43% had Medicaid, and 21% had Medicare. The majority of participants self-referred.

The first two groups (*N*=11, *N*=13) were led by the PI, who has extensive experience with mindfulness-based interventions. Participants attended an average of 6.1 sessions and completed 156 min of home practice of formal mindfulness skills (meditation, body scan, yoga) per week; retention rate at 16 weeks was 90%. Participants demonstrated a significant decrease in stress (*t* = 2.65, *p*=.01, *d*=1.06) and increase in mindfulness (*t*=−2.21, *p*=.04, *d*=−0.88) from baseline to 8 weeks. From baseline to 16 weeks, participants demonstrated a significant decrease in depression (*t*=2.14, *p*=.04, *d*=.84) and stress (*t*=2.61, *p*=.02, *d*=1.02) and significant increase in mindfulness (*t*=−2.71, *p*=.01, *d*=−1.06) [58].

Participants from these first two groups were invited to participate in follow-up focus groups. In the focus groups, participants reported that the group helped them to feel calm and relaxed, manage anger, and gain control of thoughts, emotions, behavior, and ability to be in the present moment. They noted that the group being in a familiar location, bus transportation support, shorter length of classes, course materials (manual and audio CD), and the group format were factors that facilitated participation. One participant stated “We, black women, always got to be ‘superwomen’ we have to be able to do everything and that brings about a lot of stress…I came because I had put so much stress on myself and this helped me understand that I have an opportunity to learn how to calm myself down and recognize what’s going on.” The focus groups confirmed that the modifications that were made to the MBSR format and surface content were acceptable [59].

The third and fourth groups (*N*=19, *N*=22) were led by a staff health educator at the FQHC who received streamlined training in the intervention. The PI, in conjunction with another experienced mindfulness teacher, developed the training protocol and manual and trained the staff. Participants attended an average of 5.9 sessions and completed 123 min of home practice of formal mindfulness skills (meditation, body scan, yoga) per week. The retention rate at 16 weeks was 85%. Participants demonstrated a significant decrease in depression (*t* =8.46, *p*<.001, *d*=.69) and stress (*t*=4.83, *p*<.001, *d*=.69) and increase in mindfulness (*t*=−4.5, *p*<.001, *d*=−.56), functioning (*t*=3.4, *p*<.001, *d*=.43), and well-being (*t*=4.48, *p*<.001, *d*=−.56) from baseline to 8 weeks. From baseline to 16 weeks, participants demonstrated a significant decrease in depression (*t*=5.51, *p*<.001, *d*=.83) and stress (*t*=5.90, *p*<.001, *d*=.95) and increase in mindfulness (*t*=−4.71, *p*<.001, *d*=.68), functioning (*t*=3.51, *p*<.001, *d*=.53), and well-being (*t*=−4.17, *p*<.001, *d*=−.69).

Overall, outcomes among participants in the experienced instructor group were comparable to those in the novice instructor group. These preliminary data provide evidence of the acceptability, feasibility, and improved depression and stress outcomes over time from the M-Body intervention led by a FQHC staff member with streamlined teacher training [60].

### Study aims

This study is a two-arm group-treated individually randomized controlled trial to test the clinical effectiveness of a mindfulness-based intervention (M-Body) on reducing depressive symptoms among socio-economically disadvantaged, predominantly Black women in an FQHC. The M-Body intervention will be led by trained FQHC staff; fidelity to the intervention, adherence, and competence will be continuously assessed. Feedback on factors relevant to the implementation of the intervention in the FQHC, including attitudes toward implementation of intervention, implementation leadership, and attitudes toward adoption and clinical effectiveness of evidence-based practices, will be obtained by convening quarterly workgroups and individual semi-structured interviews with FQHC staff and leadership.

Our specific aims and corresponding hypotheses are to:

1. Examine the effectiveness of a mindfulness-based intervention (M-Body) compared to enhanced usual care on reducing depressive symptoms over a 6-month period among racial/ethnic minority adults at a Federally Qualified Health Center (FQHC).
  *H1a: We hypothesize that participants in the M-Body arm will experience a significantly greater reduction in depressive symptoms than those in the enhanced usual care arm.*
  *H1b: We hypothesize that greater class attendance in the M-Body intervention will be associated with a greater reduction in depressive symptoms.*
2. Conduct a broad assessment of organization and individual agency factors related to the implementation of the M-Body intervention at a FQHC using a mixed methods approach.

## Methods

### Overview

Adults who demonstrate a minimum threshold of depressive symptoms will be randomly assigned to either an eight-session mindfulness group intervention or enhanced usual care (“control”). Participants will be followed for 6 months after randomization, with assessments at baseline and 2, 4, and 6 months post-baseline (Table 1). During an in-person visit at baseline, a trained research assistant will administer the clinical instruments, collect stress-related biomarker data, and oversee the completion of the self-report surveys. At follow-up, participants will complete self-report surveys and stress-related biomarker data collection during an in-person visit and an independent evaluator (trained research assistant) will contact them by phone to assess the primary outcome. The primary outcome will be depressive symptoms at 6 months post-baseline. To take advantage of the repeated outcome assessments, secondary outcomes will compare 2-, 4-, and 6-month measurements of depression, anxiety, trauma symptoms, and anger. Northwestern University’s Institutional Review Board (IRB) and the research committee of the FQHC approved the study protocol.

**Table 1:** Description of all interactions between participants and M-BODY personnel

Assessment location	Study timeline^a^	M-BODY personnel	Measurements	Time (minutes)
In-person^b^ or phone	Pre-baseline (screening)	Research assistant or study coordinator Study phlebotomist	PHQ-9	10
In-person^b^	Baseline	Research assistant or study coordinator Study phlebotomist	MINI Self-report surveys^d^ Dried blood spot Body Mass Index Blood pressure	90
In-person^b^	2 months	Research assistant or study coordinator Study phlebotomist	Self-report surveys^d^ Dried blood spot Body Mass Index Blood pressure	90
Phone	2 months	Independent evaluator^c^	IDS-C LIFE^e^	15
In-person^b^	4 months	Research assistant or study coordinator Study phlebotomist	Self-report surveys^d^ Dried blood spot Body mass index Blood pressure	90
Phone	4 months	Independent evaluator^c^	IDS-C LIFE^e^	15
In-person^b^	6 months	Research assistant or study coordinator Study phlebotomist	Self-report surveys^d^ Dried blood spot Body Mass Index Blood pressure	90
Phone	6 months	Independent evaluator^c^	IDS-C LIFE^e^	35-45

*Abbreviations: M-BODY* mindfulness-based intervention, *NNHSC* Near North Health, *PHQ-9* Patient Health Questionnaire-9, *MINI* Mini International Neuropsychiatric Interview, *IDS-C* Inventory of Depressive Symptomatology, Clinician Administered, *LIFE* Longitudinal Interview Follow-up Evaluation

^a^All study visits were scheduled within a 2-week window of the study milestone to accommodate participants’ schedules

^b^All in-person interactions happened at an NNHSC site

^c^Independent evaluators were clinical psychology graduate students or post-doctoral fellows

^d^Self-report surveys: See Table 3

^e^LIFE is a follow-up to the MINI interview

### Study setting

This study will be conducted in partnership with the Near North Health Services Corporation, a group of eight FQHC’s in Chicago, IL, USA. The two primary sites for recruitment are accessible via public transportation. They are centrally located on a bus route. The FQHC network has approximately 46,130 patient visits per year; the majority of patients are racial/ethnic minority (71% Black, 23% Hispanic), uninsured (43.2%), and living at or below the poverty line (74%).

#### Recruitment

Patients at the FQHC who had a positive Patient Health Questionnaire-9 (PHQ-9) screen (>5) at a recent primary care visit will be recruited to participate in the study. Initial contact will be made via postal mail, followed by phone calls and text messages describing the study and inviting participation. Additionally, the FQHC healthcare providers will provide a brochure and overview of the study to all patients who exhibit depressive symptoms and can directly refer their patients to the study team. Research staff will visit the clinic at least weekly to encourage recruitment and talk to interested participants. Self-referral will be encouraged via posted flyers and brochures within the FQHC, advertisement on social media (Facebook, Twitter, and Instagram), posts on the Near North website and Facebook page, and brochure distribution at neighborhood business.

Interested persons will be offered the opportunity to participate in the study. For self-referrals and FQHC staff referrals, verbal consent to conduct the screening and eligibility assessments will be obtained by phone; for onsite referrals, this consent will be obtained in writing. Written informed consent for all eligible study participants will be obtained in person at the first study visit (baseline assessment) by a research assistant.

#### Participant eligibility

Participants will be eligible to enroll in the study if they are between the ages of 18 and 65 years, fluent in English, and enrolled in care at a FQHC and have score greater or equal to 5 on the PHQ-9, which indicates mild depressive symptoms. Exclusion criteria will include a suicide attempt in the past 30 days; endorsement of severe suicidal thoughts on the PHQ-9 (item 9); a current meditation practice defined as four or more sessions per week; or employment at the FQHC. Mental health treatment (individual or group psychotherapy and psychiatric medication) is permitted for the duration of the trial.

### Study design

**Fig. 1:**
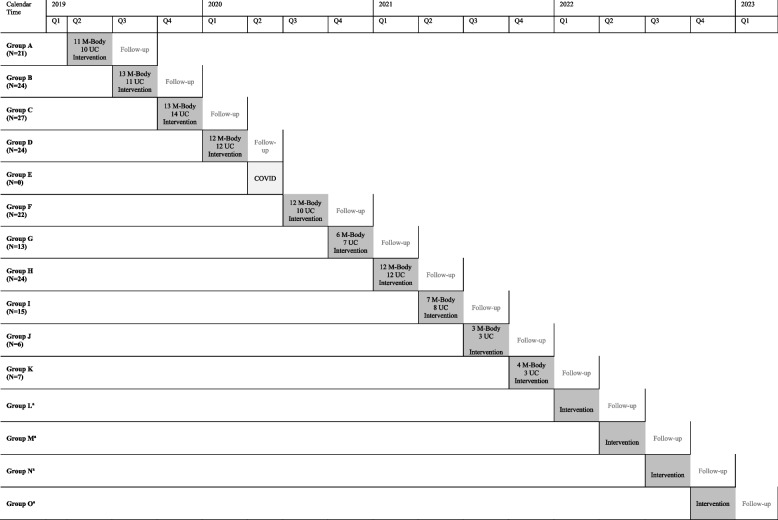
Study timeline and randomization numbers by group; M-Body, 2019–2022. ^a^Anticipated randomization waves

This study will be a block-randomized controlled trial with two arms: the M-Body intervention arm and a control arm consisting of enhanced usual care. Participants will be recruited in cohorts and randomized to conditions within cohorts; this is equivalent to a block randomized experiment with cohorts corresponding to blocks. Outcomes will be assessed at baseline and at 2, 4, and 6 months after baseline. Measurements will be obtained through a clinical interviews and completion of self-report surveys at baseline. At follow-up assessments, measures will be obtained through self-reported surveys and a phone evaluation with an independent evaluator. Figure 1 describes the intended study timeline and randomization numbers by group.

#### M-Body intervention arm

The M-Body intervention will consist of 8 weekly, 90-min group sessions led by trained FQHC staff. The M-Body curriculum will include the core content from Mindfulness-based Stress Reduction (MBSR [34];) and has been tailored to fit within the context of the FQHC setting and patient population (see Table 2 for a list of didactics). The course curriculum will include a manual and audio (CD or digital electronic file), in session and at home components. Each session will include a brief check-in, didactic, skill practice, inquiry, and review of homework. Following a skill practice, participants will engage in inquiry, a dialogue about what was noticed during the practice. Sessions will conclude by reviewing homework for the upcoming week. Participants will be asked to engage in formal home practice “as much as they can” and informal mindfulness practices. Formal practices will include sitting meditation, body scan, and hatha yoga. Informal practices will include cultivating awareness in everyday activities such as eating, walking, and communicating, noticing pleasant and unpleasant events and practicing STOP (Stop, Take Stock, Observe, Proceed). Participants will record the type and minutes of formal practice they complete and will record their informal practice on worksheets and turn it into the instructor weekly.

**Table 2:** A full list of didactics of the M-Body intervention

	Standard MBSR	FQHC adaptations
Delivery	Orientation 8 weeks, 2.5 h/week ½ day retreat	8 weeks, 90 min/week
Content	1. Simple Awareness; introduction to yoga and body scan 2. Attention and perception; introduction to yoga 3. Dealing with thoughts; noticing pleasant events 4. Stress; identifying triggers and reactions; noticing unpleasant events 5. Difficulty emotions and sensations; responding vs. reacting 6. Mindfulness & communication 7. Mindfulness & compassion 8. Developing a personal mindfulness practice	No Change
Instructor Qualifications	Attend MBSR program Complete teacher training Engage in personal mindfulness practice Has professional training and related graduate degree Embody mindfulness	Is a FQHC paraprofessional Completes condensed teacher training
Intention	To be “relevant, and accessible enough to benefit potentially anybody who might be overwhelmed by suffering…”	No Change

*Abbreviations: M-Body* Mind-Body, *MBSR* Mindfulness Based Stress Reduction, *FQHC* Federally Qualified Health Center

#### Control arm

To compare the effectiveness of the M-Body intervention against the existing real-world standard practice for depression treatment in the FQHC setting, usual care was selected as an appropriate control. The usual care to be offered here may be considered minimally enhanced because it will likely include more systematic evaluation and monitoring of depressed adults than may be typical of a community health center. Participants in this arm will receive feedback about their depression score, as well as an opportunity to ask questions of a sensitive and knowledgeable clinician. Participants will be able to contact the study team about increasing symptoms or urgent situations at any point in the study period. Participants will be provided with a brief one-page brochure on stress management skills. After their participation in the study ends, they will be invited to participate in ongoing M-Body group classes with no assessments or added incentives.

#### Intervention training

The PI will work with the FQHC leadership to identify staff who are qualified to lead the M-Body intervention. The PI and an experienced mindfulness teacher will train FQHC staff in the M-Body intervention using the same training framework established during the pilot work [60]. The instructor training was informed by the Mindfulness-based Teacher Training Program outlined by Crane et al. [61]. The instructor training includes the following: (1) Foundational training includes (a) attending an 8-week, 2.5-h-per-session MBSR course with an experienced teacher, and (b) developing a personal mindfulness practice. (2) Basic teacher training includes (a) participation in an 8-h professional training workshop led by the PI and another experienced mindfulness instructor on mindfulness theory, science, principles, pragmatics, and techniques; (b) continuing a personal mindfulness practice; (c) teaching a course; and (d) regular supervision with the PI.

All M-Body classes will be audio/video recorded. The PI will review all audio/videotapes from the previous week’s session and provide feedback to the instructor during weekly 1-h supervision. Preferred qualifications for the instructor are a minimum of a masters’ level education in psychology, social work, nursing, or other related fields. To date, two instructors were health educators, one was a licensed clinical professional counselor, one a licensed clinical psychologist, and one a licensed clinical social worker.

#### Participation incentives

All participants will be provided with single-ride public transportation cards to attend classes and study visits as needed. All study participants will receive a $20 gift card after completing the baseline assessment, and a $30 gift card after completing each of the 2-, 4-, and 6-month assessments for a total of $110 in gift cards for full participation. All M-Body participants will receive an instructional manual and audio CD, yoga mat, tote bag, and pen for use inside and outside of group sessions. Light meals will be provided at each of the in-person sessions.

### Randomization

Following consent but prior to randomization, participants will complete a clinical interview and baseline surveys, and stress biomarker data will be collected. We will then implement a block randomization procedure, with each participant in a cohort randomly assigned to 1 of the 2 arms (overall allocation ratio, 1:1). Block randomization with randomly varying block sizes was chosen to prevent prediction of assignments and thereby protect from selection bias. Randomization will be stratified by gender, initial severity of depression (Inventory of Depressive Symptomatology- Clinician Administered (IDS-C) ≥38), and receipt of treatment for depressive symptoms (antidepressants and/or psychotherapy versus none) [62]. To avoid all possibility of interference from the investigators, randomization will be fully automated, with the randomization sequence prespecified in R [63] and implemented through Research Electronic Data Capture (REDCap) [64, 65]. After randomization, research staff will provide participants with a sealed envelope containing their condition assignment. Neither the participants, study coordinators, statistical analysis team, nor the treatment providers will be blinded to treatment assignments. Independent evaluators and study co-investigators will remain blinded. To maintain the overall quality and legitimacy of the clinical trial, unblinding should occur only in exceptional circumstances when knowledge of the actual treatment is absolutely essential for further management of the participant. The Principal Investigator will discuss with the co-investigators and DSMB if she believes that unblinding is necessary.

### Retention and adherence strategies

To enhance recruitment and recognize the contributions of participants in the UC condition, these individuals will be invited to participate in the M-Body group at the end of 6 months after all data have been collected. To improve engagement in the intervention, the research staff will phone, email, or text subjects whenever they miss a session to encourage participation. Reminders will be sent to all participants to remind them of upcoming data collection and the incentives they will receive. The study team will make every effort to be flexible and accommodate participant schedules.

### Measures

For a brief overview of all measures and assessment times, see Table 3.

**Table 3:** Instruments used to collect data during the M-Body study

			Scoring			Visit administered^a^				
Instrument	Construct(s) measured	Reliability/validity^b^	No. items	Score range	Interpretation	T0	T1	T2	T3	T4
*Primary outcome*										
Structured interview guide for the inventory of depressive symptomology (Clinician-rated) Rush et al., 1986 [62]	Depressive symptoms	High/moderate Rush et al., 1986 [62]	30	0–84	0–13 = None 14–25 = Mild 26–38 = Moderate 39–48 = Severe 49–84 = Very Severe		x	x	x	x
*Secondary outcomes*										
Generalized anxiety disorder - 7-item scale Spitzer et al., 2006 [66]	Generalized anxiety	High Spitzer et al., 2006 [66]	7	0–28	5 = Mild 10 = Moderate 15 = Severe ≥10 indicates probable GAD diagnosis		x	x	x	x
Anger self-report questionnaire Reynolds et al., 1994 [67]	Anger	High/moderate Reynolds et al., 1994 [67]	30	30–180	Higher scores indicate increased trait anger		x	x	x	x
Posttraumatic stress disorder (PTSD) for DSM-5 (PCL-5) Weathers, et al., 2013 [68]	Trauma; PTSD symptom severity	High Blevins et al., 2015 [69]	20	0–80	≥31 indicates probable PTSD diagnosis		x			x
*Other psychological assessments*										
Patient Health Questionnaire-9-item Kroenke et al., 2001 [70]	Depression	High Kroenke et al., 2001 [70]	9	0–27	1–4 = Minimal 5–9 = Mild 10–14 = Moderate 15–19 = Moderately severe 20–27 = Severe	x				
Mini International Neuropsychiatric Interview Sheehan et al., 1998 [71]	Major depressive episode(s), Dysthymia, Generalized Anxiety Disorder	High Sheehan et al., 1998 [71]	46	N/A	N/A		x			
Longitudinal Interval Follow-up Evaluation – II (Modified) Keller et al., 1987 [72]	Major Depressive Episode(s), Dysthymia, Generalized Anxiety Disorder	High Keller et al., 1987 [72]	22	0–48	A coding of 5 and above indicate meeting DSM-IV criteria.			x	x	x
HIV cost and services utilization study baseline questionnaire, module 6: utilization of care—modified Shapiro et al., 1999 [73]	Mental health providers, social support, usual source of medical care, utilization of care and services used	N/A	44	N/A	N/A		x	x	x	x
Social problems questionnaire (Modified) Corney and Clare 1985 [74]	Social problems, difficulties and dissatisfactions	N/A	28	0–112	Higher scores indicate greater social difficulties		x			
Perceived stress scale Cohen et al., 1983 [75]	Perceived helpfulness and perceived self-efficacy	High/moderate Cohen et al., 1983 [75]	10	0–40	Higher scores indicate greater perceived stress		x	x	x	x
Life events checklist for DSM-5 (LEC-5) Weathers, et al., 2013 [76]	Exposure to events known	High Gray et al., 2004 [77]	17	N/A	N/A		x			x
Criterion A extended assessment (from PCL-5 with LEC-5 and Criterion A) Weathers, et al., 2013 [68]	PTSD Criterion A stressor information	N/A	9	N/A	N/A		x			x
Five facet mindfulness questionnaire Baer et al., 2006 [78]	Mindfulness: Observing, describing, acting with awareness, non-judging of inner experience, non-reactivity to inner experience	High Baer et al., 2006 [78]	39	39–195	Higher scores indicate greater trait-like mindfulness tendencies		x	x	x	x
Reflection and rumination questionnaire Trapnell and Campbell, 1999 [79]	Rumination and reflection	High Trapnell and Campbell, 1999 [79]	24	12–60	Higher scores indicate increased rumination		x	x	x	x
Difficulties in emotion regulation scale—short form Kaufman et al., 2016 [80]	Emotion regulation: non-acceptance of emotional response. Difficulties engaging in goal-directed behavior, impulsive control difficulties, lack of emotional awareness, limited access to emotion regulation strategies, lack of emotional clarity	High Kaufman et al., 2016 [80]	18	18–90	Higher scores indicate greater difficulties with emotion regulation		x	x	x	x
Cognitive emotion regulation questionnaire – short form Garnefski et al., 2001 [81]	Cognitive emotion: self-blame, other-blame, ruination, catastrophizing, putting into perspective, positive refocusing, positive reappraisal, acceptance and planning	High/moderate Garnefski et al., 2001 [81]	18	18–90	Higher scores indicate greater use of cognitive strategy		x	x	x	x
Self-compassion scale—short form Raes et al., 2011 [82]	Self-compassion: Self-kindness, self-judgment, common humanity, isolation, mindfulness, over-identified	Moderate Raes et al., 2011 [82]	12	12–60	Higher scores reflect greater self-compassion		x	x	x	x
Spirituality scale Delaney, 2003 [83]	Spirituality; spiritual beliefs, intuitions, lifestyle choices, practices, and rituals	High Delaney, 2003 [83]	23	23–138	Higher scores reflect greater reported experience of spirituality		x			
*Implementation measures*										
Implementation Questionnaire	Attitudes toward implementation of intervention	N/A	8	N/A			x			
Implementation Leadership Scale (Modified) Aarons et al., 2014 [84]	Implementation leadership	High Aarons et al., 2014 [84]	7	0–48			x	x	x	x
Evidence-Based Practice Attitude Scale Aarons, 2004 [85]	Attitudes toward adoption of evidence-based practices	High Aarons, 2004 [85]	15	0–60			x			
Clinical Effectiveness and Evidence-Based Practice Questionnaire (Modified) Upton and Lewis, 1998 [86]	Professional’s attitudes toward concepts and application of evidence-based practice and clinical effectiveness	High Upton and Lewis, 1998 [86]	3	0–24			x	x	x	x

*Abbreviations: No.* number of, *HIV* human immunodeficiency virus, *DSM-5* Diagnostic and Statistical Manual of Mental Disorders, 5^th^ Edition, *DSM-IV* Diagnostic and Statistical Manual of Mental Disorders, 4^th^ Edition

^a^The visit abbreviations “T0,” “T1,” “T2,” “T3,” and “T4” correspond to screening, baseline, and 2-, 4-, and 6-month follow-ups

^b^Low (*α* < 0.60, *κ* < 0.41, ICC < 0.50, *r* < 0.35), moderate (0.60 < *α* < 0.70, 0.41 < *κ* < 0.60, 0.50 < ICC < 0.75, 0.36 < *r* < 0.67), high (*α* > 0.70, *κ* > 0.60, 0.75 > ICC > 0.90, *r* > 0.90) (100, 101, 102, 103)

#### Primary outcome

Depressive symptoms, measured 6 months after baseline, will be the primary outcome of this study, assessed using the Inventory of Depressive Symptomatology- Clinician Administered (IDS-C) [87]. The IDS-C is a 30-item scale that was administered by an independent evaluator who is blind to study condition at baseline and 2, 4, and 6 months. The IDS-C has high internal consistency with a Cronbach’s alpha of .92 [87]. The Longitudinal Interval Follow-up Evaluation – II (LIFE) will be used to assess symptoms at follow-up periods [72].

#### Secondary outcomes

Major depressive disorder and persistent depressive disorder will also be assessed as a secondary outcome using The Mini International Neuropsychiatric Interview (MINI 6.0) at *baseline* [71]. The MINI is a short clinician-administered diagnostic interview that assesses current and lifetime psychiatric disorders based on the American Psychiatric Association’s Diagnostic and Statistical Manual of Mental Disorders – 4^th^ edition (DSM-IV) and International Classification of Diseases (ICD-10) [71]. Study team members administering the MINI were trained by an expert clinician and rated mock interviews until they reached acceptable reliability with the expert clinician.

Longitudinal measures of depressive symptoms, generalized anxiety, anger, and trauma symptoms will also be secondary outcomes (Table 3). Generalized anxiety will be measured using the Generalized Anxiety Disorder-7 Scale (GAD-7) [66]. The GAD-7 is a 7-item self-report measure of generalized anxiety symptoms. Scores of 10 or greater are predictive of a likely GAD diagnosis. Anger will be measured using the Anger Self-Report Questionnaire, which is a single-factor, 30-item self-report measure of anger [67]. Both anxiety and anger will be assessed by self-report at baseline and 2, 4, and 6 months. Post-traumatic stress will be measured using the PTSD Checklist for DSM-5 (PCL-5) [68]. The PCL-5 is a 20-item self-report measure of PTSD symptom severity, administered at baseline and 6 months.

#### Process outcomes

M-Body group audio/video recordings will be used in intervention fidelity assessments. Coders will review the audio/video recordings of the M-Body sessions and assess fidelity, adherence, and competence using the Mindfulness-based Interventions – Teaching and Assessment Criteria scale [88]. Fidelity assessments will be completed for each instructor, each session, for every group for the duration of the study. These data will be used to monitor fidelity to intervention delivery and improve staff training.

Over the course of the M-Body study, all stakeholders will complete measures to assess implementation readiness, feasibility, and fidelity. Stakeholders will include the FQHC Medical Director, Director of Behavioral Health, Director of Quality Assurance Improvement, Instructors, and any other FQHC staff who will be directly involved in the research study. A complete list of implementation measures can be found in Table 3.

#### Covariates, moderators, and mediators

A secondary aim of this study is to investigate M-Body effects on specified biological, psychological, and socio-environmental mechanisms. To this end, we will collect data in three realms: social and environmental factors, psychological outcomes, and stress-related biomarkers.

We will consider age, socioeconomic characteristics, and social and environmental factors as potential moderators of the intervention effects. Socioeconomic characteristics will include education (high school diploma or less versus some college or more), employment (unemployed, part-time, full-time), and income (below versus above the federal poverty line). Social and environmental factors will be assessed at study baseline using (1) the Social Problems Questionnaire (SPQ) as a proxy for environmental stress; (2) spirituality; (3) social support; and (4) a summary of traumatic life events via the PTSD Checklist for DSM-5 with Life Events Checklist for DSM-5 (LEC-5) and Extended Criterion A as a proxy for long-term stress.

Potential mediators will include the following: (1) mindfulness measured via Five Facet Mindfulness Questionnaire (FFMQ); (2) stress, measured via Perceived Stress Scale; (3) Cognitive Regulation measured via Cognitive and Emotion Regulation Questionnaire (CERQ-SF); (4) Emotion Regulation measured via Difficulties in Emotion Regulation Scale (DERS-SF); (5) Reflection and Rumination measured via Reflection and Rumination Questionnaire; (6) Self-Compassion via Self Compassion Scale; and (7) stress-related inflammatory biomarkers, including c-reactive protein (CRP), interleukin-6 (IL-6), tumor necrosis factor-α (TNF-α), and IL-10.

Stress-related inflammatory biomarkers will be analyzed using dried blood spot (DBS) samples. Trained study personnel will collect one to five drops of free-flowing capillary blood on standardized filter paper of the same grade used for neonatal screening using a disposable and sterile micro-lancet. Samples will be covered and left to dry overnight and then frozen at −30 °C until analyzed. Samples will be analyzed for CRP using a high-sensitivity enzyme-linked immunosorbent assay (ELISA) previously validated for DBS. IL-6, TNF-α, and IL-10 will be quantified using a highly sensitive multiplex electrochemiluminescent immunoassay that allows for the simultaneous quantification of each cytokine in DBS. The protocol has excellent lower limits of detection (0.2 to 0.7 pg/mL), precision (intra-assay CVs<7.5%), and reliability (inter-assay CV<9%) and high correlations between matched plasma and DBS samples across the entire assay range. We will be applying standard laboratory quality control procedures, including running samples in duplicate, inspecting calibration curves for fit and day-to-day variation, and measuring quality control samples with each assay.

#### Data collection, quality control, and confidentiality

Data handling procedures for data transfer, entry, and maintenance will be performed by trained study personnel under the supervision of Northwestern Information Technology Services. Self-reported data will be collected via online assessment directly into a Northwestern University-hosted REDCap database. We will collect information at every stage of recruitment, randomization, and intervention so that we can report patient flow according to the CONSORT (CONsolidated Standards Of Reporting Trials) guidelines [89].

#### Protection of human participants and assessment of safety

This trial will be monitored for safety by an independent Data and Safety Monitoring Board (DSMB), composed of two professors of psychology and one psychiatrist with expertise in randomized controlled trials, mood disorders, women’s mental health, and mindfulness interventions. Protocol violations, including inadequate or delinquent informed consent, failure to satisfy inclusion criteria, improper unblinding, incorrect or missing baseline assessments, mishandled samples, materially inadequate record keeping, missed primary outcome data collection, intentional deviation from protocol, Good Clinical Practice (GCP), or regulations by study personnel, and repeated protocol non-compliance will be reported to the Northwestern IRB immediately. Serious adverse events (SAEs), including psychiatric hospitalization determined to be study related, major adverse cardiac events (stroke, myocardial infarction), and death, will be reported to the IRB by filing a report on the Northwestern IRB website. The trial will be stopped if the DSMB determines that there is a risk of SAEs in the intervention arm; in this case, the DSMB has power to recommend study continuation, modification, or termination the study.

Spirit reporting guidelines are being followed in the conduct of this trial [90].

### Statistical plans

#### Sample size

Our sample size was chosen to ensure adequate power to detect significant differences in the primary study outcome, IDS-C, between the intervention arm and enhanced usual care. We plan to enroll both sexes, but we anticipate 85% of participants to be female; thus, power considerations are stratified by sex. The project will enroll 274 adults (226 women, 48 men; ages 18–65 years) who meet previously described eligibility criteria.

To determine study sample size, we assumed a two-sided 5% type-I error rate, standard deviation of up to 14 units for the IDS-C (according to preliminary data on the IDS-self report), and a meaningful difference across arms of six points on average on the IDS-C [58]. Analyses will use linear mixed effects models with random effects for blocks and fixed effects for treatment assignment. Since participants will be enrolled in groups of approximately 15 at a time and intervention will be delivered in a group setting, there is a possibility of an intra-cluster correlation (ICC). Previous literature has suggested that ICC tends to be low in group intervention studies, so here we considered ICC values 0.001–0.01 [91]. Under these assumptions, eight groups of women per arm (each of size 12; i.e., 96 women per arm) or 192 women total would ensure 84% power to detect a meaningful difference across arms. To combat anticipated 15% study attrition, we plan to enroll 226 women [59]. Power analyses were conducted via simulation in the R programming language (version 4.1.1) and verified using PASS (version 15) [92]. Note that our initial design and power analyses involved cluster randomization rather than block randomization. Because cluster randomized trials tend to have lower power, our original power analyses suggested 240 women would be required for 80% power, and thus, opting for block randomization reduces the number of participants required.

Additionally, we plan to enroll two groups of men, each of size approximately 12 (i.e., 48 men). We do not anticipate that we will have adequate power to detect meaningful differences across arms in men, and thus, analyses in men will be hypothesis-generating and assess feasibility in this population.

Though our power is greater than 80% for our primary aim, we anticipate an ultimate sample size of 192 women provides 80% power to detect small to moderate standardized effect sizes (*d*≥0.4) in secondary outcomes under the same assumptions. With respect to correlation coefficients (since partial correlation coefficients will play a role in mediation analyses), we will have 80% power to detect a correlation coefficient of 0.2 at the 5% level in either the overall sample or just in women, and a correlation coefficient of 0.39 or larger in men.

#### Statistical analyses

Proportions, means, medians, and ranges will be used to summarize baseline distributions of demographic factors and clinical mental health presentation (i.e., baseline depression, anxiety, trauma, stress) at baseline stratified by study arm. Exploratory data analyses will be conducted wherein individual trajectories of each primary and secondary study outcome over 6 months by study arm are visualized.

##### Primary outcome analyses

We intend to conduct all primary statistical analyses according to the intention-to-treat (ITT) principle. We will estimate the average treatment effect for participants classified by study arm, independent of participation in any M-Body classes. This approach is conservative because it protects the effect of randomization from confounding introduced by subject dropout and crossover. We will employ both a classic ITT analysis as well as a modified ITT analysis, wherein all participants completing at least one follow-up visit will be included, regardless of adherence. This will allow us to understand the bias introduced by study dropout.

The primary outcome analysis will consist of a linear mixed model (LMM) in which mean 6-month IDS-C will be compared across study arms (fixed effect), controlling for gender, baseline IDS-C scores, and baseline receipt of psychotherapy, the variables upon which randomization is stratified. We will include a random group effect as this is an individually randomized group-treated design. This approach will account for within-group associations to more precisely estimate intervention effects. The coefficient for study arm will address the question: “for two patients with the same baseline depressive symptoms, gender, and baseline receipt of psychotherapy, if one is given the M-Body intervention and the other enhanced usual care, will the patients have different depressive symptoms after 6 months?”

As a sensitivity analysis, we will estimate the average treatment effect among the treated. This analysis will allow us to estimate among individuals randomized to the intervention whether there is a dose-effect relationship between the number of classes attended and depressive symptoms. This analysis, done only in the intervention arm, will consist of a LMM in which 6-month IDS-C will be regressed upon the number of M-Body sessions attended (fixed effect), controlling for gender, baseline IDS-C scores, and baseline receipt of psychotherapy, and as before, including a random group effect. The coefficient on number of sessions attended will address the question: “on average, what is the difference in six-month IDS-C that attending one more M-Body session affords, holding gender, baseline IDS-C score, and baseline receipt of psychotherapy constant?”

##### Secondary outcome analyses

We will conduct four exploratory secondary outcome analyses, one each for depression, anxiety, anger, and trauma, designed to leverage the longitudinal nature of outcome assessment in this study. The first will consist of a LMM in which IDS-C will be compared across study arms at each follow-up visit (three fixed effects), controlling for gender, baseline IDS-C scores, and baseline receipt of psychotherapy. Here we will include random effects for group participation and individual, as we will need to account for within-person correlation. We will consider a simpler model for parsimony including follow-up visit as a continuous variable, as in a linear growth mixed effects model, and select the model that minimizes the Bayesian Information Criterion. The visit-specific coefficients on study arm will address the question: “for two patients with the same baseline depressive symptoms, gender, and baseline receipt of psychotherapy, if one is given the M-Body intervention and the other enhanced usual care, will the patients have different depressive symptoms after 2, 4, and/or 6 months?” The secondary outcome analyses for GAD and anger will use similar models to those described here for IDS-C, this time additionally including baseline GAD or anger scores as appropriate. The secondary outcome analysis for trauma will consist of a linear mixed model (LMM) in which mean 6-month trauma will be compared across study arms (fixed effect), controlling for gender, baseline IDS-C scores, and baseline receipt of psychotherapy, with a random group effect. If issues with model convergence are encountered, we will consider simpler models, such as a linear model with fixed effects for groups, or a linear model with no group effects specified. No interim analysis is planned or will take place; we do not anticipate study developments to require interim analyses.

### Protocol amendments

Modifications to the protocol which may impact the conduct of the study, including changes of study objectives, study design, patient population, sample size, and study procedures, will require a formal amendment to the protocol. Such amendment will be agreed upon by the investigative team, discussed with the NIMHD program officer, and approved by the Northwestern University IRB.

### COVID-19 modifications

#### Setting

The COVID-19 pandemic began during active recruitment, enrollment, and follow-up of study participants. All in-person research activities including recruitment, screening, baseline and follow-up assessments, and M-Body classes have been conducted virtually since March 2020 using text, email, phone calls, and virtual tele-conference. For the foreseeable future, all study activities will be held virtually, in accordance with social distancing guidelines put in place by local governing officials. If state and city ordinances indicate that it is again safe to meet in person, we will re-evaluate the feasibility of returning to in-person study activities.

#### Recruitment

All recruitment activities from group 5 onwards were conducted virtually, using the electronic health records obtained via Alliance Chicago (https://alliancechicago.org/mission-history/), who maintains the electronic health record for the Near North Health Service Centers (Fig. 1). Using a curated patient list, potential participants were contacted by the research team via text, phone call, and email. As a part of receiving care at Near North, patients consent to their data being used for research purposes. Information about the study was placed on the health center’s website as well as social media outlets including Facebook and Twitter. Potential participants were also referred by their providers and word of mouth.

#### Measurements

Baseline clinical interviews that were previously administered in person (MINI and IDS-C) are now being administered via phone by a member of the research team (Table 1). Follow-up assessments that were previously conducted with an independent evaluator by phone are being administered in the same manner. Self-report surveys are distributed via a REDCap link by email/text depending on participant preference to be completed on their own within a two-week time frame. Collection of dried blood spot samples, blood pressure, and body mass index (BMI) have been temporarily paused until it is safe to resume in-person data collection.

#### Intervention

All M-Body group sessions are being conducted using Zoom. The research staff contacts all participants before the first session to assist them with the set up and functionality of Zoom. Additionally, a staff member is available to assist participants with the operationality of Zoom during group sessions, if needed. Participants are given the option to have their camera on or off. All Zoom sessions are recorded and reviewed by the PI for adherence to the protocol.

#### Study materials

Study materials including the manual and tote bag are mailed to the participants before the first session. Participants in the UC condition also receive a mailing containing key study dates and a stress reduction tip sheet.

#### Payments

Participants receive their stipend for participation using an electronic gift card. They are emailed instructions on how to access the gift card and also provided with a YouTube tutorial. If a participant is unable to access the gift card or has a preference not to have an electronic gift card, a physical gift card is sent to them in the mail.

#### Statistical analysis

The statistical analysis plan will remain largely unchanged. In principle, using group effects in our models will sufficiently account for the differences between groups, including changes in the delivery of the intervention due to COVID-19. That said, if there are any analyses where including group effects is not feasible or plausible, we will consider adding a fixed effect to our modeling indicating whether or not the group was conducted during COVID-19. Additionally, any analysis using dried blood spot data will be considered exploratory as our sample size is limited because DBS collection was paused during this time.

### Dissemination

Throughout the duration of the study, newsletters will be disseminated to health center partners and patients providing updates on study activities. Upon completion of data collection and data analyses, study results will be disseminated to participants, collaborators, and the community at large via newsletters, scientific publications, and presentations at local and national conferences. Topics for presentation or publications, contributing authors, and authorship order will be discussed among the investigative team, research team, and community health center partners. We plan to make our study protocol public and have registered all hypotheses and analyses on ClinicalTrials.gov. In addition, we will make patient-level data and analytic code available upon reasonable request and in accordance with all local, state, federal, and institutional restrictions.

### Trial status

This comparative effectiveness trial is ongoing and began recruiting participants on February 18, 2019, according to the protocol available at ClinicalTrials.gov. As of October 21, 2022, 232 individuals had completed all baseline assessments and were randomized to either M-Body or enhanced usual care. By the conclusion of data collection in June 2023, we anticipate that we will have held 16 M-Body courses, and enrolled and randomized 274 individuals (226 women and 48 men).

## Discussion

The proposed research offers an innovative integrative approach to depression treatment that is likely to be more accessible and acceptable among socio-economically disadvantaged, racial/ethnic minority adults. We are examining the effectiveness of a mindfulness-based intervention, which has demonstrated efficacy for improving psychological outcomes in the general population but has not been sufficiently examined in racial/ethnic minority groups. Additionally, are conducting a broad assessment of organizational and individual agency factors related to preparation and implementation of the M-Body intervention in a Federally Qualified Health Center (FQHC) that serves low-income, predominantly racial/ethnic minority adults. This research will be used to develop a generalizable model for delivery of streamlined mental health interventions in community settings that will be broadly disseminated and scalable to wider populations.

This study has some limitations that should be acknowledged. We are collaborating with a FQHC network in Chicago that may not be generalizable to FQHCs nationwide. To follow, it is possible that the effectiveness of the implementation of the intervention within this specific FQHC will be impacted by the unique characteristics of our partnering organization. Additionally, we are recruiting a population that experiences a greater burden of psychosocial stressors. While we are collecting some data on social problems and traumatic stress events, this does not cover in totality the stressors (i.e., exposure to violence, neighborhood chaos) experienced by our participants. These factors are likely to impact not only their engagement in the study but also psychological outcomes. Finally, the study is likely to be influenced by organizational changes that are occurring within the system. However, the strong relationship we have with our FQHC site will allow us to anticipate and account for potential changes, for example, in federal and state health care and mental health service policies that potentially, health center funding, and staff turnover.

Despite these limitations, we hypothesize the M-Body intervention will be effective in reducing depression symptoms among low-income Black women. Additionally, we anticipate receiving strong buy-in from our collaborators at the FQHC to recruit and patients for participation and train health center staff to deliver the intervention. Should we find that the intervention is effective in improving symptoms of stress, and in turn depression, and can effectively be implemented within the FQHC context, it will be a scalable model for broad implementation of culturally tailored mental health interventions in settings where the need has been extreme and access to services severely limited.

## Data Availability

The statistical analysis team (study biostatistician JMS, statistical analyst KBZ) will have access to the final trial dataset, as well as the study principal investigator IBZ and the project coordinator EZ. There are no contractual agreements that limit access for investigators.

## Abbreviations

M-body: Mind-Body
CRP: C-reactive protein
IL-6: Interleukin-6
IL-1β: Interleukin-1β
TNF-α: Tumor Necrosis Factor-α
FQHC: Federally Qualified Health Centers
MBSR: Mindfulness-Based Stress Reduction
MBCT: Mindfulness-Based Cognitive Therapy
PHQ-9: Patient Health Questionnaire-9
PI: Principal Investigator
STOP: Stop, Take Stock, Observe, Proceed
REDCap: Research Electronic Data Capture
CONSORT: Consolidated Standards of Reporting Trials
IDS-C: Inventory of Depressive Symptomatology—Clinician Administered
MINI: Mini International Neuropsychiatric Interview
GAD-7: Generalized Anxiety Disorder-7 Scale
PCL-5: PTSD Checklist for DSM-5
SPQ: Social Problems Questionnaire
LEC-5: Life Events Checklist for DSM-5
FFMQ: Five Facet Mindfulness Questionnaire
CERQ-SF: Cognitive and Emotion Regulation Questionnaire—Short Form
DERS-SF: Difficulties in Emotion Regulation Scale—Short Form
DBS: Dried blood spot
ELISA: Enzyme-linked immunosorbent assay
DSMB: Data and Safety Monitoring Board
GCP: Good Clinical Practice
SAEs: Serious adverse events
ICC: Intra-cluster correlation
ITT: Intention-to-treat
LMM: Linear mixed model
BMI: Body mass index
LIFE: Longitudinal Interview Follow-Up Evaluation
LEC-5: Life Events Checklist for DSM-5
No.: Number of
HIV: Human immunodeficiency virus
DSM-IV: Diagnostic and Statistical Manual of Mental Disorders, 4^th^ edition
DSM-5: Diagnostic and Statistical Manual of Mental Disorders, 5^th^ edition
